# Exercise Modality Affects Older Adult CT-Derived Muscle and Bone Loss During Caloric Restriction

**DOI:** 10.1093/geroni/igab046.300

**Published:** 2021-12-17

**Authors:** Ashley Weaver, Diana Madrid, Katelyn Greene, Michael Walkup, Walter Ambrosius, Anthony Marsh, W Jack Rejeski, Kristen Beavers

**Affiliations:** 1 Wake Forest School of Medicine, Winston Salem, North Carolina, United States; 2 Wake Forest University, Winston-Salem, North Carolina, United States; 3 Department of Health and Exercise Science, Wake Forest University, North Carolina, United States; 4 Wake Forest University, Winston Salem, North Carolina, United States

## Abstract

Caloric restriction (CR) can exacerbate muscle and bone loss. We examined 18-month changes in computed tomography (CT)-derived trunk muscle, and volumetric bone mineral density (vBMD) and finite element-estimated bone strength of the spine and hip in 55 older adults randomized to CR alone or CR plus aerobic (CR+AT) or resistance (CR+RT) training. Trunk muscle area loss trended higher with CR+AT [−16.8 cm^2^ (95% CI: −26.4, −7.1) vs CR: -6.7 (−12.8, −0.5), CR+RT: −9.0 (−14.5, −3.4)]. Spine vBMD loss trended higher with CR+AT [−0.014 g/cm^3^ (−0.027,−0.001) vs. CR: −0.005 (−0.022,0.012), CR+RT: −0.004 (−0.019,0.011)], and similarly for vertebral bone strength. Hip vBMD losses trended lower with CR+RT [−0.015 g/cm^3^ (−0.024,−0.006) vs. CR: −0.027 (−0.036,−0.019), CR+AT: −0.029 (−0.037,−0.020)]. Hip vBMD and trunk muscle losses were positively correlated (r=0.53), and spine vBMD loss tended to increase with trunk muscle loss (r=0.21) and fat infiltration (r=0.17). Collectively, aerobic training was less effective at preserving muscle-bone health during CR.

